# Skeletal muscle proteins important for work capacity are altered with type 2 diabetes — Effect of 10‐20‐30 training

**DOI:** 10.14814/phy2.14681

**Published:** 2021-01-10

**Authors:** Thomas Baasch‐Skytte, Thomas P. Gunnarsson, Matteo Fiorenza, Jens Bangsbo

**Affiliations:** ^1^ Department of Nutrition, Exercise and Sports University of Copenhagen Copenhagen Denmark

**Keywords:** antioxidant defense, high‐intensity interval training, ion handling, mitochondria

## Abstract

The study examined whether men with type 2 diabetes exhibit lower expression of muscle proteins important for exercise capacity, and whether exercise training promotes adaptations in these proteins. In a cross‐sectional and longitudinal study, conducted at the University of Copenhagen. Twelve men with type 2 diabetes (T2D) were compared to eleven nondiabetes counterparts (ND) matched for age and body composition (body fat percentage). T2D underwent 10 weeks of high‐intensity interval exercise training (10‐20‐30 training). T2D had lower expression of SOD1 (−62%; *p* < 0.001) and ETC complex V (−34%; *p* = 0.003), along with higher expression of ETC complex IV (+66%; *p* = 0.007), MFN2 (+62%; *p* = 0.001), and DRP1 (+30%; *p* = 0.028) compared to ND. T2D had higher (*p* < 0.001) expression of Na^+^/K^+^ α1 (+98%), α2 (+114%), and NHE1 (+144%) than ND. In T2D, training increased exercise capacity (+9%; *p* < 0.001) as well as expression of SOD2 (+44%; *p* = 0.029), ETC complex II (+25%; *p* = 0.035), III (+52%; *p* = 0.041), IV (+23%; *p* = 0.005), and V (+21%; *p* = 0.035), CS activity (+32%; *p* = 0.006) as well as Na^+^/K^+^ α1 (+24%; *p* = 0.034), Kir6.2 (+36%; *p* = 0.029), and MCT1 (+20%; *p* = 0.007). Men with type 2 diabetes exhibited altered expression of a multitude of skeletal muscle proteins important for exercise capacity. Ten weeks of 10‐20‐30 training upregulated expression of muscle proteins regulating antioxidant defense, mitochondrial function, and ion handling while enhancing exercise capacity in men with type 2 diabetes.

## Graphical abstract

Men with type 2 diabetes exhibited altered the expression of a multitude of skeletal muscle proteins important for exercise capacity. Ten weeks of 10‐20‐30 training upregulated expression of muscle proteins regulating antioxidant defense, mitochondrial function, and ion handling while enhancing exercise capacity in men with type 2 diabetes.

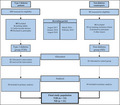

## 1 INTRODUCTION

Exercise capacity and maximum oxygen uptake (${\dot{V}O}_{2}max$) are lower in type 2 diabetes patients than in age‐ and/or weight‐matched nondiabetes controls (Baldi et al., 2003,; Fang et al., 2005; Regensteiner et al., 1998). The lower exercise capacity observed in type 2 diabetes patients may be related to both central (i.e., cardiovascular) and peripheral (i.e., muscular) factors (Baldi et al., 2003,; Reusch et al., 2013). Specifically, skeletal muscle from type 2 diabetes patients is characterized by increased oxidative damage (Aouacheri et al., 2015; Odegaard et al., 2016; Pan et al., 2010), mitochondrial dysfunction (Lowell & Shulman, 2005; Rovira‐Llopis et al., 2017), and possibly impaired ion handling (Juel et al., 2004). However, it remains unclear whether type 2 diabetes‐related muscle alterations are due to the aberrant expression of proteins involved in antioxidant defense and ion handling.

Oxidative stress, defined as an imbalance between production and removal of reactive oxygen species (ROS), while being implicated in the development of type 2 diabetes (Henriksen et al., 2011), may inhibit exercise capacity by affecting contractile function (Allen et al., 2008; Persson et al., 2019). Diabetes appears to cause alterations in proteins regulating antioxidant defense in rats (Wohaieb & Godin, 1987) and is associated with lower activity of the antioxidant enzyme superoxide dismutase (SOD) in human plasma (Pan et al., 2010; Ramakrishna & Jailkhani, 2008), implying a connection between type 2 diabetes and aberrant antioxidant defenses. Mitochondria are among the main sources of ROS in skeletal muscle (Szendroedi et al., 2011), and overwhelming mitochondrial ROS generation may lead to mitochondrial dysfunction. Accordingly, mitochondrial dysfunction has been proposed to play a critical role in the pathophysiology of type 2 diabetes (Szendroedi et al., 2011). Thus, while the interplay between aberrant antioxidant defense and mitochondrial dysfunction appears to play a critical role in the pathophysiology of type 2 diabetes and the associated impairments in exercise capacity, it remains unknown whether these features are associated with alterations in muscle proteins regulating redox homeostasis and mitochondrial function.

Skeletal muscle ion‐handling capacity plays a major role in fatigue development (Allen et al., 2008; Hostrup & Bangsbo, 2017). The pronounced Na^+^ and K^+^ perturbations occurring during exercise are caused, in part, by the ATP‐sensitive potassium channel (Kir6.2) and are counteracted by a multitude of ion‐handling proteins, including the Na^+^/K^+^‐pumps and their upstream regulator phospholemman (FXYD1). Animal studies indicate that diabetes and insulin treatment decrease and increase, respectively, muscle protein expression as well as activity of Na^+^/K^+^‐pumps (Kjeldsen et al., 1987; Schmidt et al., 1994,; Nishida et al., 1992). On the other hand, data in humans are inconsistent, with studies showing lower (Schmidt et al., 1994), higher (Djurhuus et al., 2001), or similar (Dela et al., 2004) expression of Na^+^/K^+^‐pump subunits in type 2 diabetes patients compared to nondiabetes counterparts. Besides Na^+^ and K^+^ perturbations, contractile function, and thereby exercise capacity, may be limited by rising intracellular levels of H^+^ (Allen et al., 2008). The monocarboxylate transporter subunit 1 (MCT1) and 4 (MCT4) and the Na^+/^H^+^ exchanger 1 (NHE1) contribute to regulating H^+^ handling in skeletal muscle. Interestingly, muscle protein expression of MCT1, but not MCT4 and NHE1, has been reported to be lower in type 2 diabetes patients compared to age‐ and body weight‐matched subjects (Dela et al., 2004; Juel et al., 2004). Taken together, data on ion‐handling proteins in type 2 diabetes patients are inconsistent, thus, warranting further investigations to elucidate whether type 2 diabetes and the associated lower exercise capacity is linked to aberrant expression of ion‐handling proteins.

High‐intensity interval training (HIIT) is effective in improving health‐related outcomes in patients with cardiovascular and metabolic diseases (Baasch‐Skytte et al., 2020; Fiorenza et al., 2018; Rognmo et al., 2012; Tjonna et al., 2008), and whether skeletal muscle protein adaptations are associated with improvements in exercise capacity.

Thus, the aim of this study was to investigate whether men with type 2 diabetes exhibit lower expression of skeletal muscle proteins regulating either antioxidant defense, mitochondrial function, or ion handling compared to nondiabetes counterparts and to explore the effect of HIIT on these muscle proteins. We hypothesized that men with type 2 diabetes would exhibit lower expression of proteins regulating antioxidant defense, mitochondrial function, and ion handling compared to age‐ and body composition‐matched nondiabetes counterparts. Furthermore, we hypothesized that a period of HIIT would increase the expression of these proteins while improving exercise capacity in men with type 2 diabetes.

## 2 METHODS

### 2.1 Study design

The study was part of a larger study investigating the effect of HIIT on glycemic control in men with type 2 diabetes (Baasch‐Skytte et al., 2020). In addition, for baseline comparisons nondiabetes counterparts were included from a previous study (Fiorenza et al., 2018). The present study was designed as a cross‐sectional and longitudinal study. The training intervention consisted of high‐intensity interval cycling training three times per week for 10 weeks, adhering to the 10‐20‐30 training principle (Gunnarsson and Bangsbo, 2012). Biopsies were obtained from m. vastus lateralis at rest before and after the training intervention in men with type 2 diabetes. The nondiabetes counterparts did not complete the training intervention, but had a muscle biopsy obtained at rest. The study was approved by the ethics committee of the capital region of Denmark (H‐4‐2014‐100), adheres to guidelines of the most recent version of the Declaration of Helsinki, and is registered at Clinical‐trials.gov (NCT03349944). Subjects provided oral and written informed consent prior to inclusion in the study. The manuscript is aligned with STROBE standards.

### 2.2 Subjects

**FIGURE 1:**
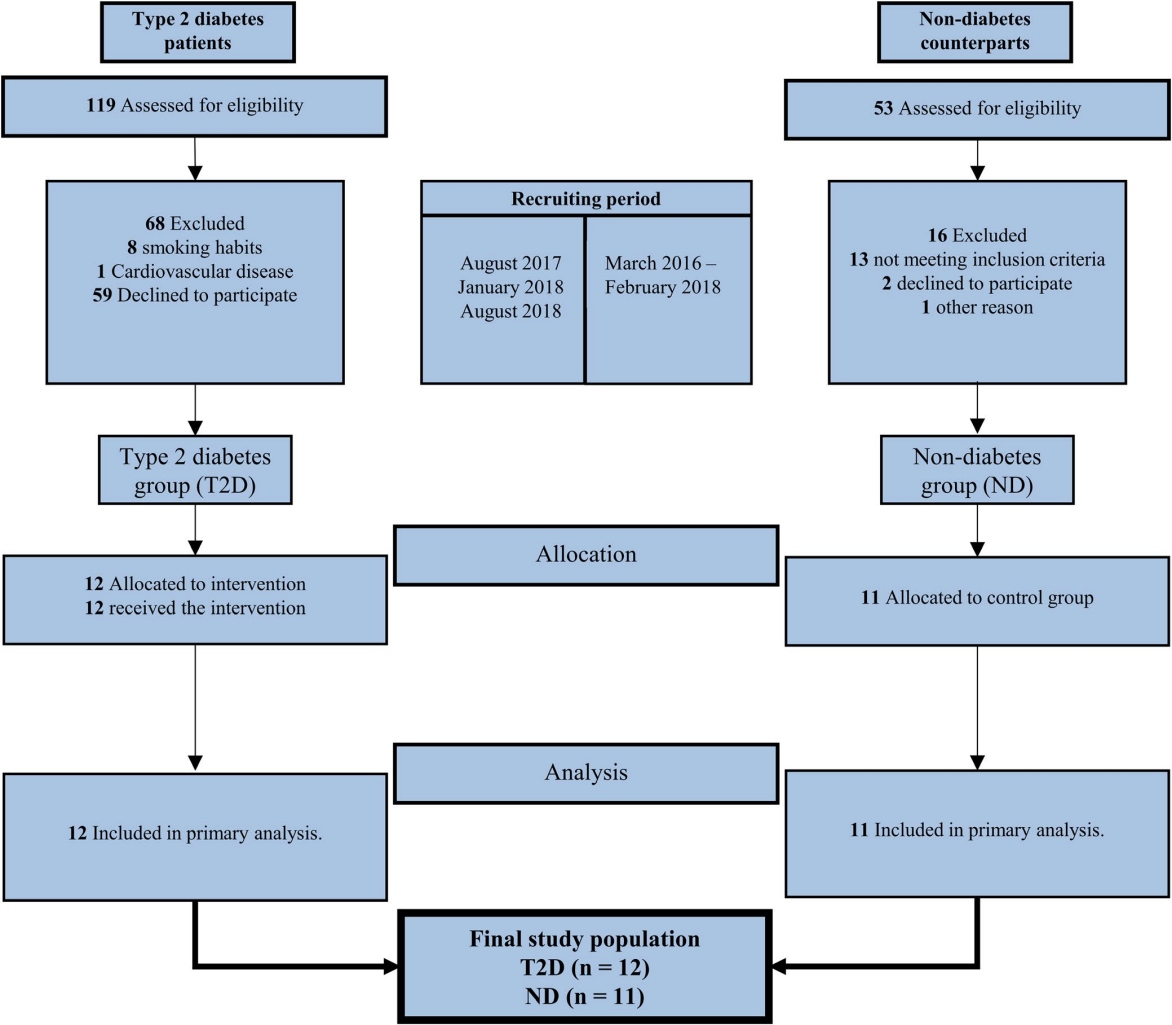
Flowchart of subjects through the study

Subjects were recruited via newspaper ads and the Danish Diabetes Association. Twenty‐three men were included; 12 inactive men with type 2 diabetes (T2D: *n* = 12) and 11 inactive nondiabetes men (ND: *n* = 11) (Table 1; Figure 1). Groups were matched based on age (±5 years) and body composition (fat%; ±3%). In T2D, one subject was newly diagnosed with type 2 diabetes (6‐12 months), five subjects were diagnosed for 1–2 years and six subjects were diagnosed for 4–15 years. Ten out of the 12 subjects included in T2D received antidiabetic medications (Table 1).

**TABLE 1:** Characteristics of the type 2 diabetes (T2D) and age‐ and body composition‐matched nondiabetes (ND) individuals

	ND (*n* = 11)	T2D (*n* = 12)	T2D (*n* = 12)	Between‐group difference (ND vs. T2D Pre)	Within‐ group difference (T2D Pre vs. T2D Post)
		Pre	Post	*p* value	*p* value
Age, years	60.3 ± 4.6	58.0 ± 3.7	58.0 ± 3.7	0.797	
Height, cm	181 ± 4.2	180 ± 3.7	180 ± 3.7	0.565	
Weight, kg	86.1 ± 6.7	91.4 ± 10.2	90.6 ± 18.5	0.510	0.858
BMI, kg/m^2^	26.1 ± 1.7	28.0 ± 2.4	27.7 ± 4.3	0.297	0.791
Fat%, %	28.7 ± 2.9	27.9 ± 4.8	27.5 ± 6.0	0.694	0,544
FFM, kg	58.9 ± 4.3	64.2 ± 5.3	64.7 ± 9.6	0.124	0.879
HbA_1c_, mmol/mol	36.4 ± 1.2	55.8 ± 8.1^†††^	51.3 ± 1.4	<0.001	0.147
HbA_1c_, %	5.5 ± 0.1	7.2 ± 0.7^†††^	6.7 ± 0.8	<0.001	0.170
Cholesterol, mmol L^−1^	5.8 ± 0.4	4.3 ± 0.6^††^		0.001	
HDL, mmol L^−1^	1.5 ± 0.2	1.3 ± 0.2		0.224	
LDL, mmol L^−1^	3.7 ± 0.3	2.2 ± 0.6^††^		0.001	
Triglycerides, mmol l^−1^	1.8 ± 0.6	2.7 ± 0.7		0.079	
${\dot{V}O}_{2}max$, ml min^−1^	3208 ± 281	2748 ± 476	2885 ± 425	0.155	0.091
${\dot{V}O}_{2}max$, ml kg^−1 ^min^−1^	37.3 ± 2.0	29.9 ± 3.1^††^	31.9 ± 2.9*	0.001	0.036
Exercise capacity, W	289 ± 29	211 ± 34^††^	229 ± 36***	0.004	<0.001
T2D diagnose, years		5.3 ± 2.7			
Antidiabetic medications, *n*		10/12			
Biguanide, *n*		8/12			
Liraglutide, *n*		1/12			
DPP‐IV inhibitor, *n*		2/12			
SGLT‐2 inhibitor, *n*		1/12			

Data are presented as mean ± SD.

Abbreviations: BMI, body mass index; Fat%, fat percentage; FFM, fat‐free mass.

^††^ Different (*p* < 0.01) from ND.

^†††^ Different (*p* < 0.001) from ND.

* Different (*p* < 0.05) from T2D pre.

*** Different (*p* < 0.001) from T2D pre.

Prior to inclusion in the study, potential subjects went through a standardized medical examination including a questionnaire regarding lifestyle, physical activity, and use of medication, a resting 12‐lead ECG, fasting blood samples for evaluation of HbA_1c_ and blood cholesterol and a graded exercise test on a bike ergometer (Monark Ergomedic 839E).

Inclusion criteria for the men with type 2 diabetes were 40‐75 years of age, type 2 diabetes diagnosis for >6 months, stable medication for >3 months. Inclusion criteria for nondiabetes counterparts were 40‐75 years of age and no regular medicine treatment. Exclusion criteria for all Subjects were >2 h of physical activity per week (daily commute by bike was accepted); evidence of renal, liver, cardiovascular and/or neuromuscular disease (except hypertension in T2D), body mass index <20 and >40 kg/m^2^, smoking and excess alcohol consumption (>14 units/week).

### 2.3 Experimental setup

Two experimental days, separated by 48 h, were conducted in T2D and ND. In addition, in T2D, the two experimental days were repeated after the training intervention within 48‐72 h after the last training session. Subjects refrained from caffeine, alcohol, and exercise for 24 h prior to the experimental days.

#### 2.3.1 Experimental day 1

Subjects reported to the laboratory in the morning after an overnight fast. After 10 min of supine rest, subjects were scanned with whole‐body dual‐energy X‐ray absorptiometry (DXA). Thereafter, a graded exercise test was performed on a mechanically braked cycle‐ergometer to determine the maximum oxygen uptake (${\dot{V}O}_{2}max$) and exercise capacity (incremental peak power output; (W). The test consisted of two 4‐min warm‐up bouts at a submaximal intensity. After 3 min of passive recovery, an incremental ramp test was performed starting at 80 W and with increments of 20 W min^−1^ until volitional exhaustion. During the test, pulmonary gas exchanges were measured breath‐by‐breath using an online gas analysis system (Oxycon Pro, Viasys Healthcare, Hoechberg, Germany). ${\dot{V}O}_{2}\max$ was determined as the highest value achieved during a 30 s period. Criteria used for achievement of ${\dot{V}O}_{2}max$ were a plateau in ${\dot{V}O}_{2}$ despite an increase in workload and a respiratory exchange ratio above 1.10.

#### 2.3.2 Experimental day 2

Subjects reported to the laboratory in the morning after an overnight fast. After 15 min of rest in the supine position, a 3 mm incision was made over the lateral portion of the thigh under local anesthesia (2 ml lidocaine without epinephrine, 20 mg/ml Xylocain; AstraZeneca Pharmaceuticals), and a biopsy was obtained from m. vastus lateralis using a percutaneous Bergstrom needle with suction.

#### 2.3.3 Training intervention

The intervention consisted of high‐intensity interval cycling training three times per week for 10 weeks, adhering to the 10‐20‐30 training principle (Gunnarsson and Bangsbo, 2012). The 10‐20‐30 training consists of five consecutive 1‐min exercise periods divided into 30, 20, and 10 seconds at low (≈30–100 W), moderate (≈60–180 W), and maximal (≥400 W) intensity (data not included). Subjects controlled the intensity by a brake force and self‐chosen cadence, but were instructed to increase the load (brake force and/or cadence) from the 30‐ to the 20‐s intervals, and to perform “all‐out” during the 10‐s sprints. Subjects were verbally encouraged to push as hard as possible during each 10‐20‐30 training session. Training sessions were conducted on Body Bikes (model HBS, Denmark) in a nonclinical local municipality setting (fitness center in Copenhagen, Denmark) by trained personnel. Prior to each training session, subjects conducted a 10‐min low‐intensity warm‐up before completing 3 × 5 min of 10‐20‐30 training interspersed by 2 min of passive recovery. Heart rate was measured beat by beat during training sessions throughout the intervention period using Polar heart rate monitors (Polar Team^2^, Kempele, Finland).

### 2.4 Measurements and analysis

#### 2.4.1 Body composition

Whole‐body fat‐free mass, fat mass, and fat percentage were measured by DXA (Lunar iDXA; GE Healthcare), as previously described (Baasch‐Skytte et al., 2020). The scanner was calibrated prior to measurements in accordance with the manufacturer's guidelines. All scans were conducted by the same operator.

#### 2.4.2 Muscle samples

Muscle samples were snap‐frozen in liquid N2 and stored at −80°C. The samples were weighed before and after freeze‐drying to determine water content. Visible fat, connective tissue, and blood were dissected away under standardized conditions (22°C and 30% relative humidity). After dissection, 2.5 and 1.5 mg of tissue was stored in separate tubes for analysis of muscle protein content and maximal enzyme activity of citrate synthase, respectively. Protein content and enzymatic activity were determined in duplicates, that is, two different samples were obtained from the same muscle specimen and the mean value of the two samples was used as a result.

#### 2.4.3 Muscle protein content

Protein content was determined in muscle lysates through SDS‐PAGE and western blot analysis. First, the freeze‐dried and dissected muscle sample was homogenized for 2 × 30 s at 29 Hz (Qiagen Tissuelyser II, Retsch GmbH) in a fresh cold buffer (10% glycerol, 20 mM Na‐pyrophosphate, 150 mm NaCl, 50 mM HEPES (pH 7,5), 1% NP‐40, 20 mm β‐glycerophosphate, 2 mM Na_3_VO_4_, 10 mm NaF‐poison, 2 mM PMSF, 1 mm EDTA (pH 8,0), 1 mm EGTA (pH 8,0), 10 µg/ml Aprotinin, 10 µg/ml Leupeptin, 3 mm Benzamidine). After homogenization, the samples were rotated end over end for 1 h at 4°C and centrifuged for 20 min at 13,000 RPM at 4°C. Then, the supernatant was collected, and protein content was determined in triplicates using a BSA standard kit (Thermo Scientific). Lysates were diluted (double‐distilled H_2_O) to equal protein concentrations in a 6× sample buffer (7 ml of 0.5 M Tris‐base, 3 ml glycerol, 0.93 g DTT, 1 g SDS, and 1.2 mg bromophenol blue).

Equal amounts of protein were loaded in duplicates in separate wells on 4‐15% or 16.5% precast Criterion gels (Bio‐Rad Laboratories). Samples from the same participant were loaded in adjacent wells on the same gel. The same pool of a mixed human muscle standard lysate was loaded in three different wells per gel, and the mean intensity of these samples was used for normalization to allow gel‐to‐gel comparison, as previously described (Thomassen et al., 2018). No housekeeping proteins were used as loading controls, as this procedure may increase the variation in the determination of the primary protein of interest (Goasdoue et al., 2016; Li & Shen, 2013; Murphy & Lamb, 2013).

Proteins were separated according to their molecular weight via SDS‐PAGE electrophoresis and proteins were semi‐dry blotted to a polyvinylidene difluoride membrane (MilliporeSigma). The membranes were blocked in 2% skim milk or 3% BSA in a mixture of tris‐buffered saline and Tween 20 (TBST) before incubation overnight at 4°C in primary antibody diluted in either 2% skim milk or 3% BSA (Table 2). Afterward, membranes were washed in TBST and incubated in secondary antibody for 1 h at room temperature. Then, membranes were washed in TBST for 3 × 15 min. The membrane staining was visualized by incubation with a chemiluminescent horseradish peroxidase substrate (Millipore) before image digitalization (Chemi Doc MP, Bio‐Rad Laboratories). Western blot band intensities were quantified by densitometry (total band intensity adjusted for background intensity) using Image lab V.4.0 (Bio‐Rad Laboratories). All western blot analyses were performed at the same time.

**TABLE 2:** Antibodies used for western blotting analysis

Antibody	Manufacturer	Catalog nr.	RRID	Antibody solution	Protein loading amount (µg)	Gel type	Dilution
Catalase	Abcam	Ab1877	AB_302649	3% BSA	12	4‐15%	1:5000
DRP1	Cell Signaling Technology	5391	AB_11178938	3% BSA	12	4‐15%	1:1000
FXYD1	Proteintech	13721‐1AP	AB_2108296	2% milk	9	4‐15%	1:2500
Kir 6.2	Santa Cruz Biotechnology	Sc‐11228	AB_2265235	2% milk	15	4‐15%	1:500
MCT1	Santa Cruz Biotechnology	Sc‐14916	AB_2189200	3% BSA	12	4‐15%	1:250
MCT4	Millipore	AB3316P	AB_11214041	2% milk	12	4‐15%	1:1000
MFN2	Cell Signaling Technology	11925	AB_2750893	2% milk	12	4‐15%	1:1000
Nak α1	DSHB	AB‐528092	AB_528092	3% BSA	15	4‐15%	1:500
Nak α2	Millipore	07‐674	AB_390164	3% BSA	9	4‐15%	1:500
Nak β1	Thermo Fischer Scientific	MA3‐930	AB_2060990	2% milk	9	4‐15%	1:1000
NHE1	Millipore	MAB3140	AB_94707	3% BSA	12	4‐15%	1:500
OXPHOS	Abcam	110411	AB_2756818	2% milk	9	16.5%	1:500
SOD1	Millipore	574597	AB_2255005	3% BSA	12	4‐15%	1:1000
SOD2	Millipore	06‐984	AB_310325	2% milk	12	4‐15%	1:1000

#### 2.4.4 Maximal activity of citrate synthase

The muscle sample was homogenized (1:400) in a 0.3 mol/L phosphate BSA buffer adjusted to pH 7.7 and analyzed for the maximal enzyme activity of citrate synthase using fluorimetry (Fluoroscan Ascent, Thermo Scientific) as previously described (Lowry & Passonneau, 1972). Enzymatic activity was determined in duplicates from the same muscle specimen.

### 2.5 Statistics

To determine between‐group differences at baseline, an independent‐sample t‐test was used. To determine within‐group changes with the training intervention, a linear mixed model was used with time as fixed factor and participant as a random factor. Data were normal distributed, and model checking was based on Q‐Q plots and Shapiro–Wilk's test. The significance level was set at *p* < 0.05. Statistical analyses were carried out using IBM SPSS Statistics 26 (IBM) and are based on sample sizes of *n* = 12 for T2D and *n* = 11 for ND, unless stated otherwise. Data are presented as mean ± SD.

## 3 RESULTS

### 3.1 Training

**FIGURE 2:**
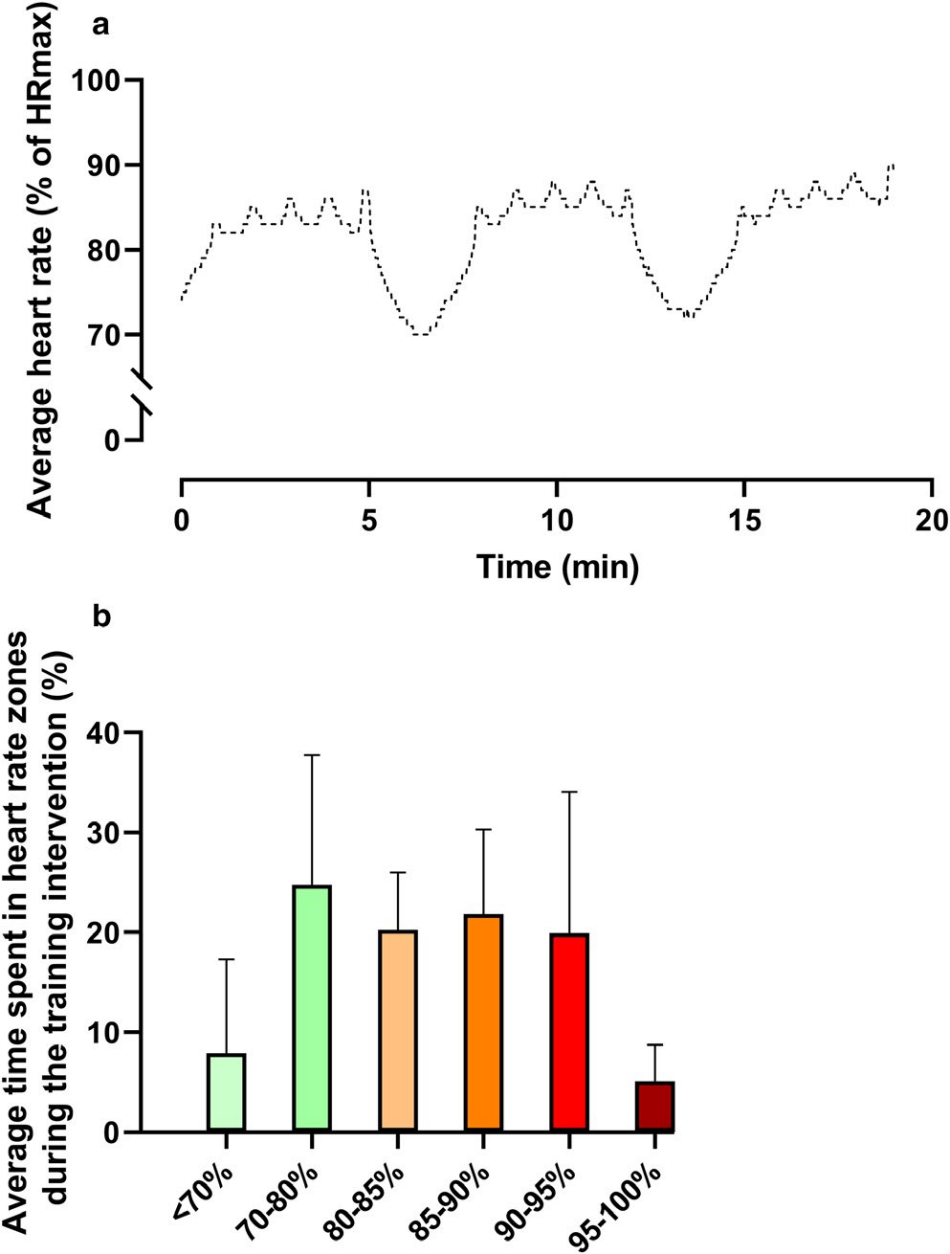
Average heart rate (excluding the 10‐min warm‐up period) (a) and average time spent in heart rate zones (b) during 10 weeks of 10‐20‐30 training in male type 2 diabetes patients (T2D; *n* = 12). Data are presented as mean ± SD

Compliance during the training intervention was 89% ± 4% in T2D corresponding to 2.7 training sessions per week. Average heart rate during training sessions was 82 ± 5% of HRmax (including rest periods), and time spent above 85% of HRmax during training was 46 ± 7% of total training time (Figure 2). No adverse events occurred during the intervention.

### 3.2 ${\dot{V}O}_{2}max$ **and exercis**e **capacity**

In T2D, ${\dot{V}O}_{2}max$ relative to body mass (ml kg^−1 ^min^−1^) was 20% lower (*p* = 0.001) than in ND, whereas absolute ${\dot{V}O}_{2}max$ was not different between groups. Exercise capacity was 27% lower (*p* = 0.004) in T2D than in ND (Table 1). In T2D, ${\dot{V}O}_{2}max$ relative to body mass increased (*p* = 0.036) by 7% (29.9 ± 3.1 vs. 31.9 ± 2.9 ml kg^−1 ^min^−1^), whereas absolute ${\dot{V}O}_{2}max$ did not change (2748 ± 476 vs. 2885 ± 425 ml min^−1^) with training. Exercise capacity increased (*p* < 0.001) by 9% (211 ± 34 vs. 229 ± 36 W) with training (Table 1).

### 3.3 Skeletal muscle protein expression

**FIGURE 3:**
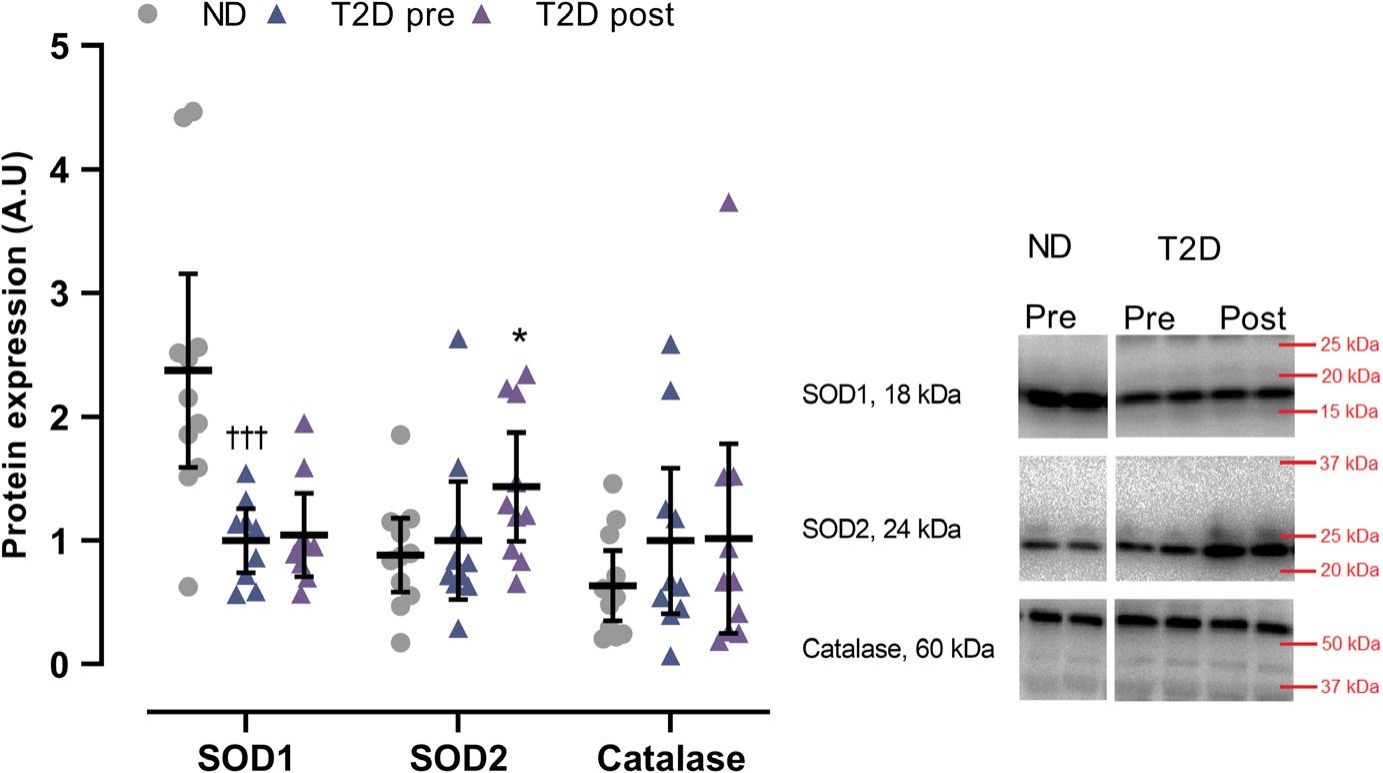
Expression of superoxide dismutase 1 (SOD1), superoxide dismutase 2 (SOD2), and catalase in male type 2 diabetes patients (T2D; SOD1; *n* = 9, SOD2; *n* = 10, Catalase; *n* = 10) before (pre) and after (post) 10 weeks of 10‐20‐30 training and in matched nondiabetes counterparts (ND, *N* = 11). Data are presented as mean ± SD including individual values. ^†††^Different (*p* < 0.001) from ND. *Different (*p* < 0.05) from T2D pre

In T2D, muscle expression of SOD1 was 62% lower (*p* < 0.001) than in ND, whereas the expression of SOD2 and catalase was not different between groups (Figure 3). In T2D, expression of SOD2 increased (*p* = 0.029) by 44% with training, whereas SOD1 and catalase expression did not change (Figure 3).

**FIGURE 4:**
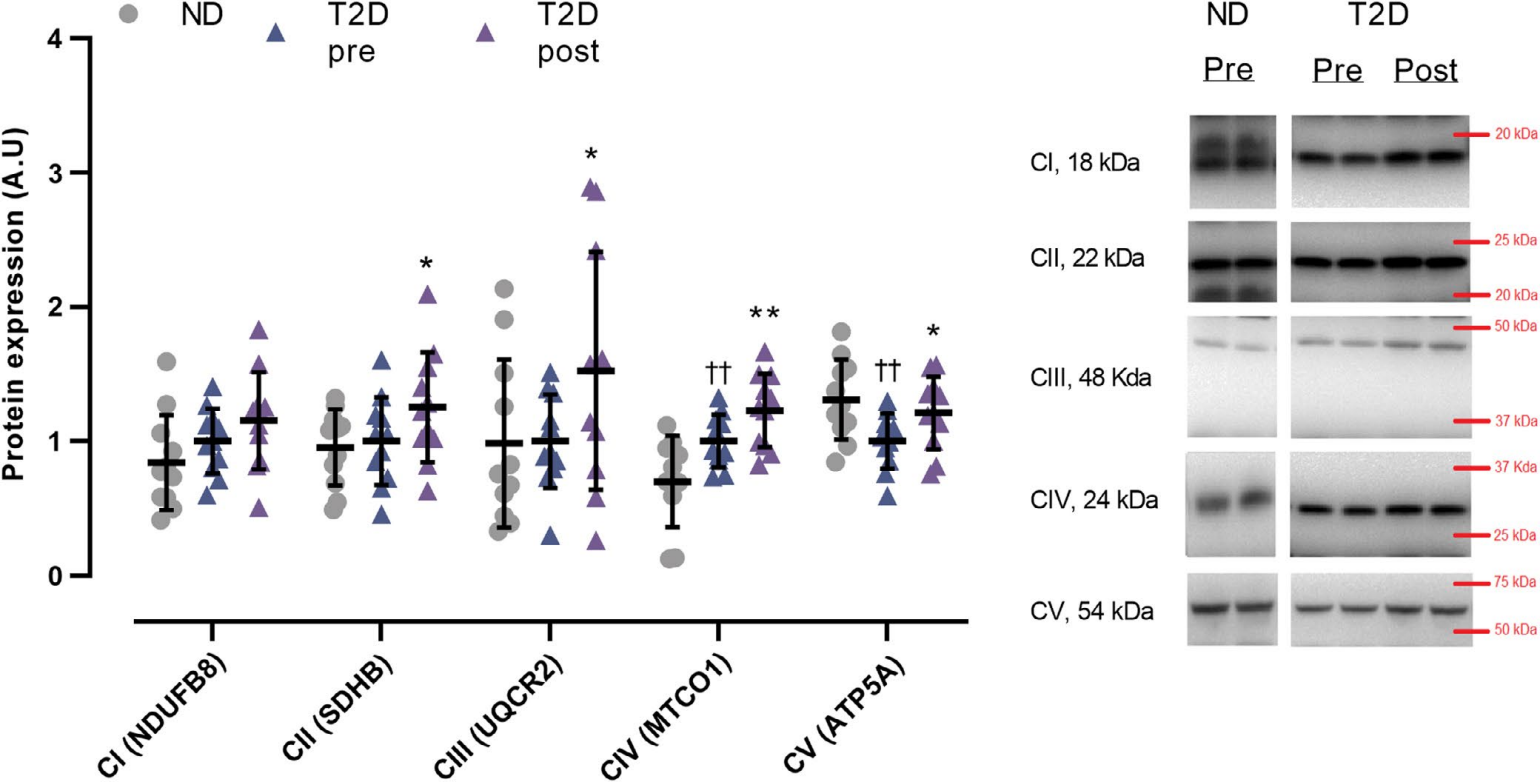
Expression of subunits of the electron transport chain (ETC) complex I–V and maximal citrate synthase (CS) activity in male type 2 diabetes patients (T2D, ETC *n* = 11, CS activity *n* = 8) before (pre) and after (post) 10 weeks of 10‐20‐30 training and in matched nondiabetes counterparts (ND, *n* = 11). Data are presented as mean ±SD including individual values. ^††^Different (*p* < 0.01) from ND. *Different (*p* < 0.05) from T2D pre. **Different (*p* < 0.05) from T2D pre

**FIGURE 5:**
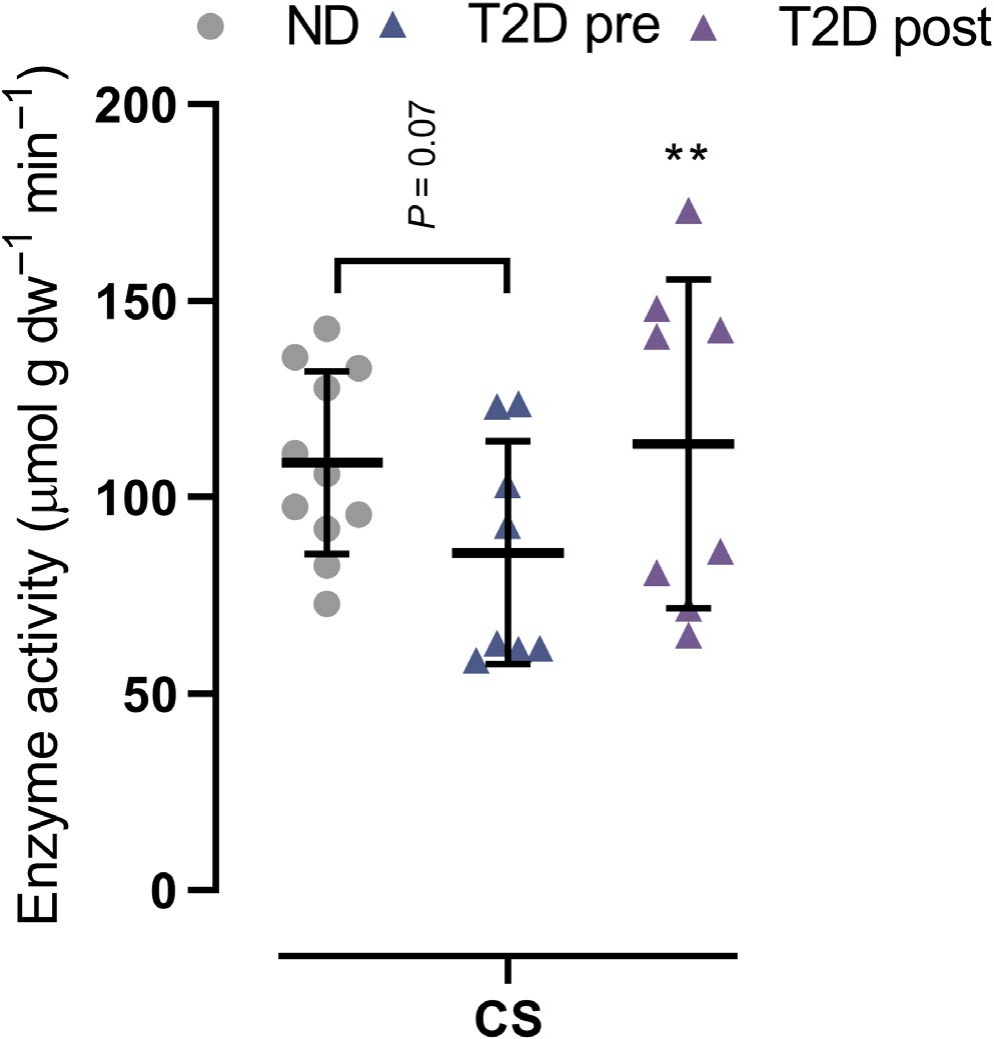
Maximal citrate synthase (CS) activity in male type 2 diabetes patients (T2D, *n* = 8) before (pre) and after (post) 10 weeks of 10‐20‐30 training and in matched nondiabetes counterparts (ND, *n* = 11). Data are presented as mean ± SD including individual values. **Different (*p* < 0.05) from T2D pre

In T2D, muscle expression of ETC complex IV was 66% higher (*p* = 0.007) than in ND, whereas complex V was 34% lower (*p* = 0.003), with no difference between groups in complex I, II, and III (Figure 4). In T2D, expression of complex II, III, IV, and V increased by 25% (*p* = 0.035), 52% (*p* = 0.041), 23% (*p* = 0.005), and 21% (*p* = 0.035), respectively, with training, whereas the expression of complex I did not change (Figure 4). Maximal activity of CS was similar (*p* = 0.07) between groups and increased (*p* = 0.006) by 32% with training (Figure 5).

**FIGURE 6:**
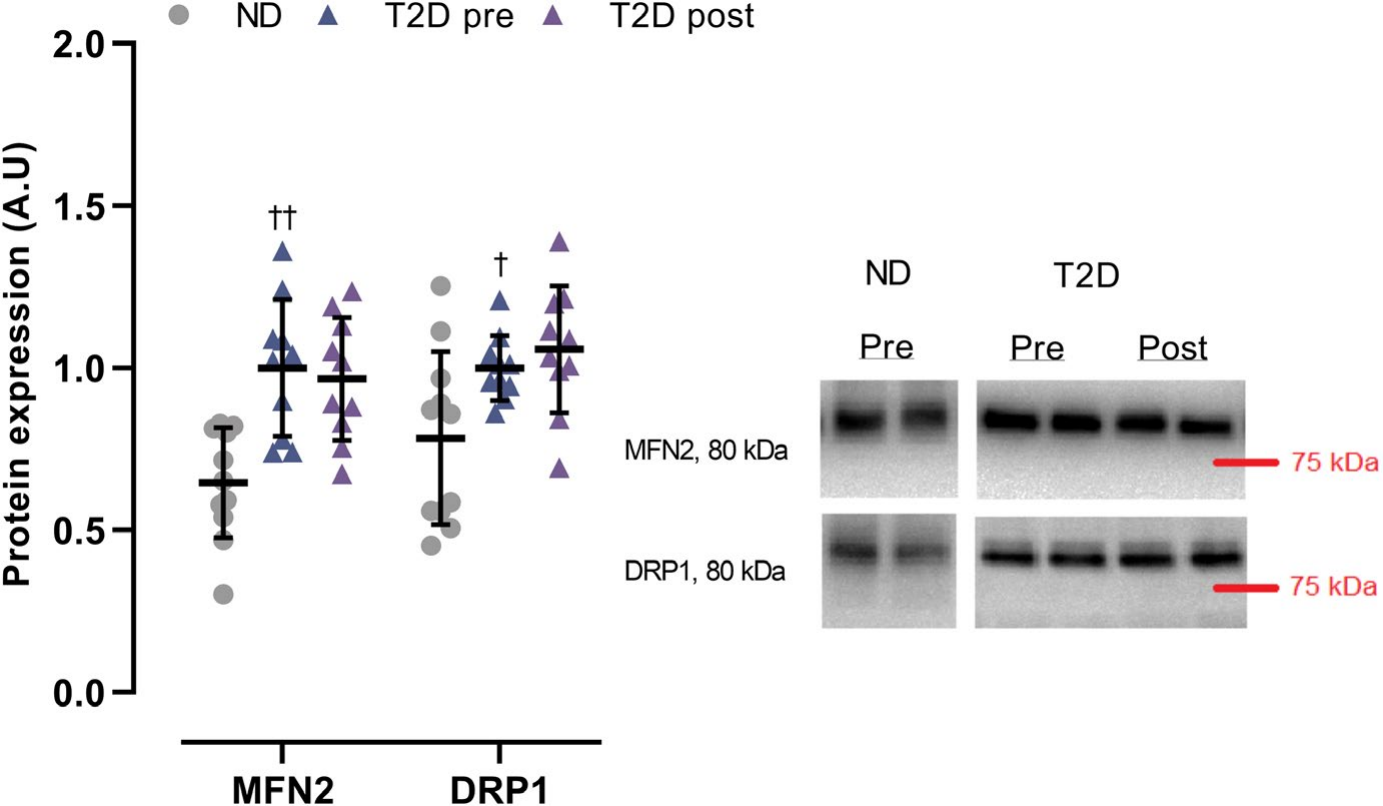
Expression of Mitofusin‐2 (MFN2) and Dynamin‐related protein 1 (DRP1) in male type 2 diabetes patients (T2D; *n* = 11) before (pre) and after (post) 10 weeks of 10‐20‐30 training and in matched nondiabetes counterparts (ND, *n* = 11). Data are presented as mean ± SD including individual values.^†^Different (*p* < 0.05) from ND.^††^Different (*p* < 0.01) from ND

In T2D, expression of muscle MFN2 and DRP1 was 62% (*p* = 0.001) and 30% (*p* = 0.028) higher, respectively, than in ND, and did not change with training (Figure 6).

**FIGURE 7:**
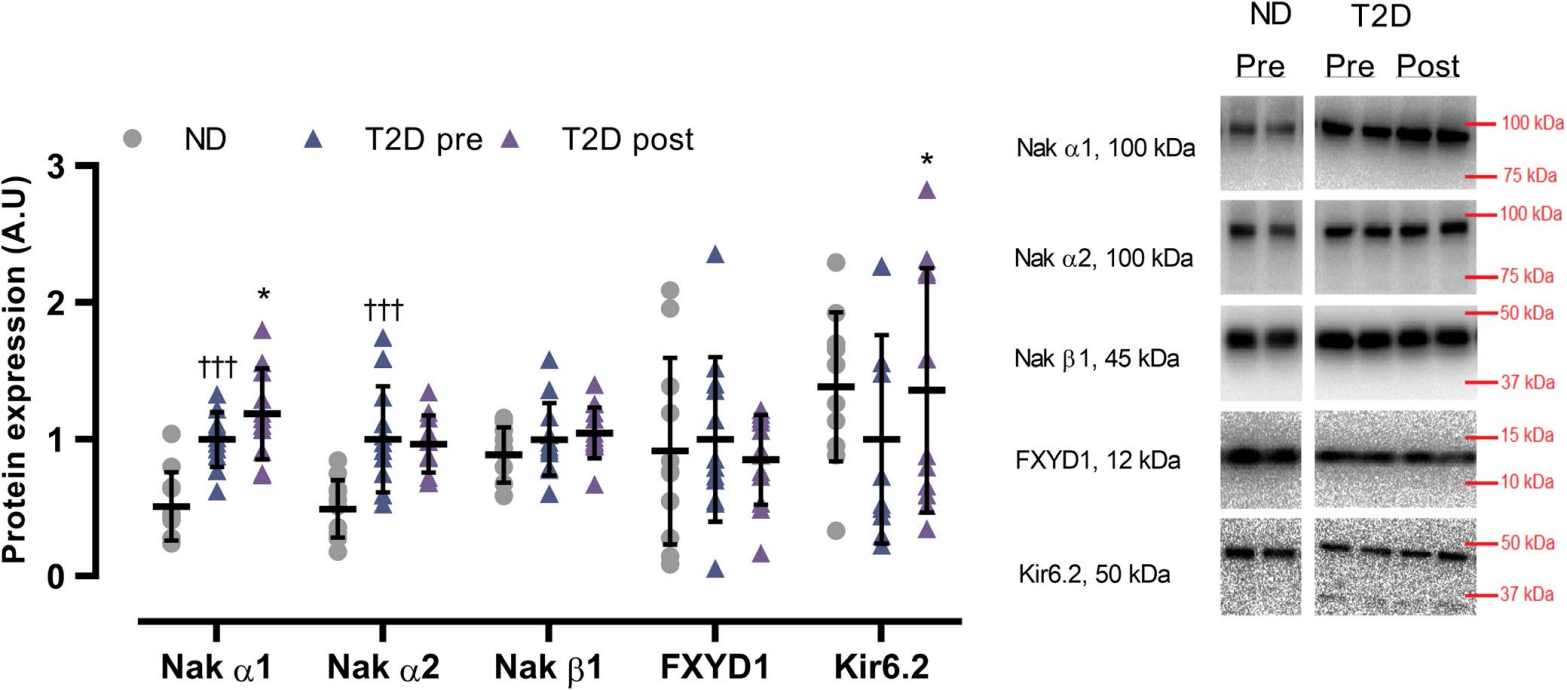
Expression of muscle Na^+^/K^+^α1, α2, and β1 subunit, Phospholemman (FXYD1), and Kir6.2 in male type 2 diabetes patients (T2D, Na^+^/K^+^α1; *n* = 11. Na^+^/K^+^α2; *n* = 12. Na^+^/K^+^β1; *n* = 12. FXYD1; *n* = 12. Kir6.2; *n* = 11) before (pre) and after (post) 10 weeks of 10‐20‐30 training and in matched nondiabetes counterparts (ND, *n* = 11). Data are presented as mean ± SD including individual values.^†††^Different (*p* < 0.001) from ND. *Different (*p* < 0.05) from T2D pre

In T2D, the expression of muscle Na^+^/K^+^ α1 and α2 was 98 and 114% higher (*p* < 0.001), respectively, than in ND, with no difference in the expression of muscle Na^+^/K^+^ β1, FXYD1, and Kir6.2 (Figure 7). In T2D, expression of muscle Na^+^/K^+^ α1 and Kir6.2 increased by 24% (*p* = 0.045) and 36% (*p* = 0.029), respectively, with training, whereas no change was observed in expression of Na^+^/K^+^α2 and β1 and FXYD1 (Figure 7).

**FIGURE 8:**
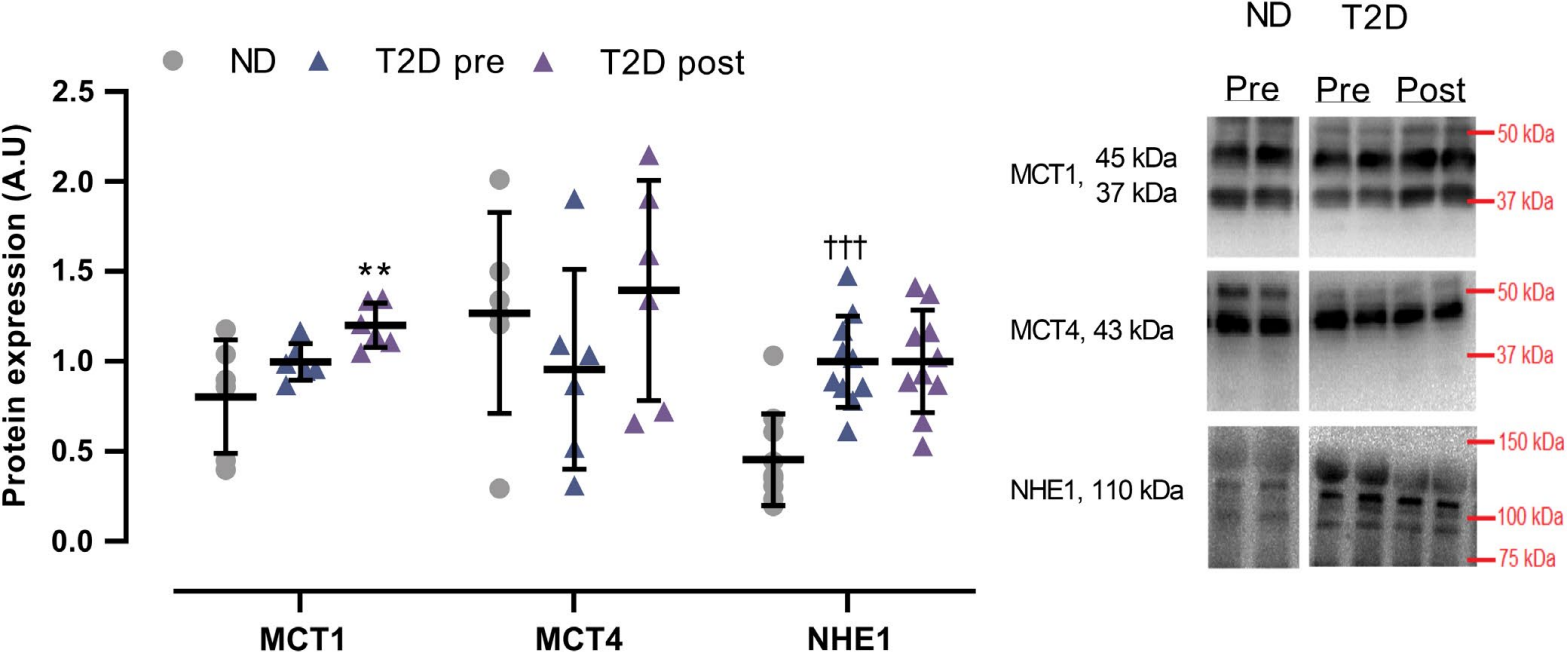
Expression of Monocarboxylate transporter 1 and 4 (MCT1, MCT4) and sodium‐hydrogen exchanger 1 (NHE1) in male type 2 diabetes patients (T2D, MCT1/4 *n* = 6; NHE1; *n* = 10) before (pre) and after (post) 10 weeks of 10‐20‐30 training and in matched nondiabetes counterparts (ND, MCT1/4 *n* = 6, NHE1 *n* = 11). Data are presented as mean ± SD including individual values. ^†††^Different (*p* < 0.001) from ND. **Different (*p* < 0.01) from T2D pre

In T2D, muscle NHE1 expression was 144% higher (*p* < 0.001) than in ND, with no difference in MCT1 and MCT4 (Figure 8). In T2D, expression of MCT1 increased (*p* = 0.007) by 20% with training, whereas NHE1 and MCT4 expression did not change (Figure 8).

## 4 DISCUSSION

The major finding of this study was that the expression of a multitude of muscle proteins important for exercise capacity were altered in men with type 2 diabetes compared to nondiabetes counterparts. Specifically, protein levels of SOD1 and ETC complex V were lower in men with type 2 diabetes. On the other hand, and in contrast to our hypothesis, men with type 2 diabetes had higher protein levels of ETC complex IV, MFN2, DRP1, Na^+^/K^+^ α1, Na^+^/K^+^ α2, and NHE1 compared to the nondiabetes counterparts. In the men with type 2 diabetes, the 10‐20‐30 cycling training intervention elevated protein expression of SOD2 and ETC complex II, III, IV, and V, maximal activity of CS, as well as protein expression of Na^+^/K^+^ α1, Kir6.2, and MCT1. Lastly, 10‐20‐30 cycling training improved exercise capacity and ${\dot{V}O}_{2}max$ relative to bodyweight. The strengths of this study are the matching of the groups (age and body composition), and that the training intervention was conducted in a nonclinical setting with a high compliance, which increases generalizability of the study outcomes. Furthermore, the weekly activity level in both groups was <2 h per week consisting of walking and occasional transport by bike, thus differences in baseline ${\dot{V}O}_{2}max$ were not caused by differences in activity level. A limitation of the study is the small sample size, which increases the risk of type 2 errors, and the lack of a training intervention in the nondiabetes group. Furthermore, only men were included which limits generalizability of study outcomes. It should also be noted that protein content does not describe the functionality of the respective proteins.

That expression of muscle SOD1, that is, the cytosolic isoform of SOD, but not SOD2, that is, the mitochondrial isoform of SOD, was lower in the men with type 2 diabetes may suggest that type 2 diabetes is associated with blunted cytosolic antioxidant protection. Our observation, while agreeing with studies reporting lower levels of total plasma SOD activity in type 2 diabetes patients compared to healthy control subjects (Pan et al., 2010; Ramakrishna & Jailkhani, 2008), is novel. The observation is the first, to our knowledge, to indicate alterations in SOD protein expression in skeletal muscle in men with type 2 diabetes compared to nondiabetes counterparts. Expression of SOD1 did not change with training in men with type 2 diabetes, whereas SOD2 increased, suggesting a training‐induced enhancement in mitochondrial antioxidant protection. In agreement, total plasma SOD activity in type 2 diabetes patients has been observed to be higher following a training intervention (Oliveira et al., 2012). Of note, however, the men with type 2 diabetes in this study did not exhibit lower catalase protein expression compared to the nondiabetes counterparts. Taken together these findings may indicate that men with type 2 diabetes have a reduced cytosolic antioxidant defense system, and that the mitochondrial antioxidant defense system is more responsive to exercise training than the cytosolic antioxidant defense system. However, further studies including assessments of antioxidant enzyme activity are required to confirm the present findings. The observed training‐induced increase in SOD2 may be due to higher mitochondrial content, as maximal activity of CS, a marker of mitochondrial content (Larsen et al., 2012), also increased with the training intervention.

The men with type 2 diabetes had lower expression of ETC complex V and higher expression of complex IV than the nondiabetes counterparts, suggesting diabetes‐related alterations in the proteins of the ETC. Lower oxidative enzyme activity (He et al., 2001) and mitochondrial respiration (Phielix et al., 2010) have been reported in type 2 diabetic subjects compared to nondiabetes controls, supporting a lower ATP production capacity in type 2 diabetic subjects. However, other studies reported normal mitochondrial function compared to healthy subjects when normalized to mitochondrial content (Boushel et al., 2007), and one study did not report a relationship between insulin sensitivity and expression of OXPHOS genes (Timmons et al., 2018). Nevertheless, the training‐induced elevations in ETC complex II, III, IV, V, and in maximal activity of CS in this study are in accordance with the adaptations observed in type 2 diabetes patients following 2 weeks of aerobic high‐intensity interval training (Little et al., 2011), possibly indicating an increased muscle mitochondrial content (Larsen et al., 2012) and pointing toward the effectiveness of high‐intensity interval training to improve muscle oxidative capacity in type 2 diabetes patients.

The higher MFN2 protein expression observed in the men with type 2 diabetes is surprising as abundance of MFN2 has been shown to correlate positively with insulin sensitivity (Bach et al., 2005) and no differences in MFN2 levels have been reported between lean and healthy as well as between obese and type 2 diabetic subjects (Gundersen et al., 2020). Also, the present findings are in contrast with a recent report showing reduced muscle MFN2 protein expression in young (<30 years) type 2 diabetes patients compared to healthy subjects (Hernandez‐Alvarez et al., 2010). However, the unequal distribution of men and women in the experimental groups in the latter study (Hernandez‐Alvarez et al., 2010) and that only men were included in this study makes comparisons between the studies difficult. Interestingly, training did not change MFN2 expression, which is in contrast to the increase in MFN2 shown in type 2 diabetes patients (Little et al., 2011) and in hypertensive subjects of similar age and body composition (Fiorenza et al., 2018) following a high‐intensity training intervention. The higher DRP1 expression observed in the men with type 2 diabetes was expected as DRP1 content has been shown to correlate positively with insulin resistance (Jheng et al., 2012), however, our results are in contrast with the absence of differences in DRP1 between lean, healthy obese and type 2 diabetic obese subjects (Gundersen et al., 2020). The lack of training‐induced changes in DRP1 expression agrees with findings in a study of old sedentary subjects (Fealy et al., 2014), but not with the observation of increased DRP1 protein content in healthy subjects with a HIIT intervention (Perry et al., 2010). This suggests that, in elderly subjects, DRP1 expression is not responsive to exercise, whereas phosphorylation of DRP1 decrease with training (Fealy et al., 2014).

The observed higher expression of muscle Na^+^/K^+^ α1 and α2 in the men with type 2 diabetes is in contrast with evidence indicating no differences between type 2 diabetes and nondiabetes subjects (Dela et al., 2004). Such discrepancy might relate to differences in the experimental design, as the groups in this study were matched not only for age and body weight but also for body composition; a factor possibly affecting ion‐handling proteins, as lower expression of Na^+^/K^+^ pumps has been observed in overweight compared to lean subjects, albeit in erythrocytes (DeLuise et al., 1982). Notably, other studies showed higher (Schmidt et al., 1994) or lower (Djurhuus et al., 2001) levels of Na^+^/K^+^ pump expression in type 2 diabetes patients compared to healthy subjects, but in neither of these studies, subjects were matched for age or body composition. The higher expression of Na^+^/K^+^ α1 and α2 subunits observed in the men with type 2 diabetes may be explained by the high plasma insulin levels associated with the progressing insulin resistance in these individuals, as insulin has been observed to increase the expression of Na^+^/K^+^ pump subunits (Hatou et al., 2010; Schmidt et al., 1994; Sweeney & Klip, 1998). An alternative explanation is that the men with type 2 diabetes may have compensated for lower muscle Na^+^/K^+^ pump activity, as suggested to occur in diabetes‐induced animal models (Nishida et al., 1992). The training‐induced increase in Na^+^/K^+^ α1 content is in line with the proposed association between the increase in α1 subunit and improvements in exercise performance (Iaia et al., 2008). Similar to Na^+^/K^+^ α1 and α2, the observation that muscle NHE1 expression was higher in the men with type 2 diabetes than in the nondiabetes counterparts is in contrast with prior evidence indicating no difference between type 2 diabetes patients and nondiabetes subjects (Dela et al., 2004). The higher expression of NHE1 may be explained by the high plasma insulin and glucose levels associated with insulin resistance, as insulin and glucose have been observed to increase the activity of NHE1 in human monocytes (Sarigianni et al., 2010). This does, however, not explain the lack of a difference in NHE1 expression previously reported (Dela et al., 2004). The men with type 2 diabetes and nondiabetes counterparts had similar expression of muscle MCT1 and MCT4; a finding partly in line with another study showing reduced expression of MCT1, but not MCT4 (Juel et al., 2004) with type 2 diabetes. As MCT1 is highly expressed in oxidative muscle fibers and MCT4 is mainly expressed in fast glycolytic muscle fibers (Wilson et al., 1998), our results suggest that the training intervention increased the H^+^ handling proteins primarily in the oxidative muscle fibers. Furthermore, the present findings indicate that glycolytic fiber‐specific adaptations in H^+^ handling may be blunted in type 2 diabetes patients, as training‐induced increments in MCT4 have been reported in healthy subjects, but not in type 2 diabetes patients (Juel et al., 2004).

The exercise capacity, determined by the peak power output achieved during the incremental cycling exercise, was lower in the men with type 2 diabetes than in the nondiabetes counterparts, confirming that type 2 diabetes is associated with impairments in physical performance. Exercise capacity increased by 9% following the training intervention, which coincided with a 7% increase in the relative ${\dot{V}O}_{2}max$ and a nonsignificant increase in 5% in absolute ${\dot{V}O}_{2}max$, implying that the improvement in ${\dot{V}O}_{2}max$ have contributed to the increased exercise capacity. The muscle adaptations with higher levels of SOD2, ETC complex II, III, IV, and V, maximal activity of CS, as well as expression of Na^+^/K^+^ α1, Kir6.2, and MCT1 with the training intervention may also have contributed to the increased exercise capacity. Nevertheless, future studies examining muscle protein adaptations with exercise training in larger cohorts of type 2 diabetes patients are warranted to unravel whether the present findings are mechanistically linked to enhancements in exercise capacity.

In summary, this study showed that men with type 2 diabetes exhibited lower expression of muscle SOD1 and ETC complex V and higher expression of ETC complex IV, MFN2, DRP1, Na^+^/K^+^ α1, and α2 and NHE1 compared to nondiabetes counterparts matched for age and body composition, which was associated with a lower exercise capacity for the men with type 2 diabetes than the nondiabetes counterparts. 10‐20‐30 training improved exercise capacity while increasing expression of SOD2, ETC complex II, III, IV, and V, maximal activity of CS, as well as expression of Na^+^/K^+^ α1, Kir6.2, and MCT1 in the men with type 2 diabetes.

## DISCLOSURE SUMMARY

JB and TPG have authored a book (in Danish) on the effects of exercise training on performance and health, including effects of 10‐20‐30 training. Otherwise, there is nothing to disclose associated with this manuscript.

## AUTHORS’ CONTRIBUTIONS

T.B.S., T.P.G., M.F., and J.B designed the study. T.B.S., T.P.G., and M.F. collected the data. T.B.S. performed the statistical analysis. T.B.S., M.F., and J.B wrote the draft manuscript. All the authors edited and approved the final version of the submitted manuscript.

## Data Availability

Data are available by request to the principal investigator.

## References

[phy214681-bib-0001] Allen, D. G. , Lamb, G. D. , & Westerblad, H. (2008). Skeletal muscle fatigue: cellular mechanisms. Physiological Reviews, 88(1), 287–332. 18195089 10.1152/physrev.00015.2007

[phy214681-bib-0002] Aouacheri, O. , Saka, S. , Krim, M. , Messaadia, A. , & Maidi, I. (2015). The investigation of the oxidative stress‐related parameters in type 2 diabetes mellitus. Canadian Journal of Diabetes, 39(1), 44–49. 25065473 10.1016/j.jcjd.2014.03.002

[phy214681-bib-0003] Baasch‐Skytte, T. , Lemgart, C. T. , Oehlenschlager, M. H. , Petersen, P. E. , Hostrup, M. , Bangsbo, J. , & Gunnarsson, T. P. (2020). Efficacy of 10–20‐30 training versus moderate‐intensity continuous training on HbA1c, body composition and maximum oxygen uptake in male patients with type 2 diabetes: A randomized controlled trial. Diabetes, Obesity & Metabolism, 22(5). 10.1111/dom.13953 31903682

[phy214681-bib-0004] Bach, D. , Naon, D. , Pich, S. , Soriano, F. X. , Vega, N. , Rieusset, J. , Laville, M. , Guillet, C. , Boirie, Y. , Wallberg‐Henriksson, H. , Manco, M. , Calvani, M. , Castagneto, M. , Palacín, M. , Mingrone, G. , Zierath, J. R. , Vidal, H. , & Zorzano, A. (2005). Expression of Mfn2, the Charcot‐Marie‐Tooth neuropathy type 2A gene, in human skeletal muscle: effects of type 2 diabetes, obesity, weight loss, and the regulatory role of tumor necrosis factor alpha and interleukin‐6. Diabetes, 54(9), 2685–2693. 16123358 10.2337/diabetes.54.9.2685

[phy214681-bib-0005] Baldi, J. C. , Aoina, J. L. , Oxenham, H. C. , Bagg, W. , & Doughty, R. N. (2003). Reduced exercise arteriovenous O_2_ difference in Type 2 diabetes. Journal of Applied Physiology, 94(3), 1033–1038. 12571134 10.1152/japplphysiol.00879.2002

[phy214681-bib-0006] Boushel, R. , Gnaiger, E. , Schjerling, P. , Skovbro, M. , Kraunsoe, R. , & Dela, F. (2007). Patients with type 2 diabetes have normal mitochondrial function in skeletal muscle. Diabetologia, 50(4), 790–796. 17334651 10.1007/s00125-007-0594-3 PMC1820754

[phy214681-bib-0007] de Oliveira, V. N. , Bessa, A. , Jorge, M. L. , Oliveira, R. J. , de Mello, M. T. , De Agostini, G. G. , Jorge, P. T. , & Espindola, F. S. (2012). The effect of different training programs on antioxidant status, oxidative stress, and metabolic control in type 2 diabetes. Applied Physiology, Nutrition and Metabolism, 37(2), 334–344. 10.1139/h2012-004 22458821

[phy214681-bib-0008] Dela, F. , Holten, M. , & Juel, C. (2004). Effect of resistance training on Na, K pump and Na+/H+ exchange protein densities in muscle from control and patients with type 2 diabetes. Pflugers Archiv. European Journal of Physiology, 447(6), 928–933. 14685860 10.1007/s00424-003-1213-x

[phy214681-bib-0009] DeLuise, M. , Rappaport, E. , & Flier, J. S. (1982). Altered erythrocyte Na+ + K+ pump in adolescent obesity. Metabolism, 31(11), 1153–1158. 7132741 10.1016/0026-0495(82)90167-6

[phy214681-bib-0010] Djurhuus, M. S. , Vaag, A. , & Klitgaard, N. A. (2001). Muscle sodium, potassium, and [(3)H]ouabain binding in identical twins, discordant for type 2 diabetes. Journal of Clinical Endocrinology and Metabolism, 86(2), 859–866. 11158058 10.1210/jcem.86.2.7239

[phy214681-bib-0011] Fang, Z. Y. , Sharman, J. , Prins, J. B. , & Marwick, T. H. (2005). Determinants of exercise capacity in patients with type 2 diabetes. Diabetes Care, 28(7), 1643–1648. 15983314 10.2337/diacare.28.7.1643

[phy214681-bib-0012] Fealy, C. E. , Mulya, A. , Lai, N. , & Kirwan, J. P. (2014). Exercise training decreases activation of the mitochondrial fission protein dynamin‐related protein‐1 in insulin‐resistant human skeletal muscle. Journal of Applied Physiology, 117(3), 239–245. 24947026 10.1152/japplphysiol.01064.2013 PMC4122691

[phy214681-bib-0013] Fiorenza, M. , Gunnarsson, T. P. , Ehlers, T. S. , & Bangsbo, J. (2018). High‐intensity exercise training ameliorates aberrant expression of markers of mitochondrial turnover but not oxidative damage in skeletal muscle of men with essential hypertension. Acta Psychologica, 225(3), e13208. 10.1111/apha.13208 30339318

[phy214681-bib-0014] Goasdoue, K. , Awabdy, D. , Bjorkman, S. T. , & Miller, S. (2016). Standard loading controls are not reliable for Western blot quantification across brain development or in pathological conditions. Electrophoresis, 37(4), 630–634. 26593451 10.1002/elps.201500385

[phy214681-bib-0015] Gundersen, A. E. , Kugler, B. A. , McDonald, P. M. , Veraksa, A. , Houmard, J. A. , & Zou, K. (2020). Altered mitochondrial network morphology and regulatory proteins in mitochondrial quality control in myotubes from severely obese humans with or without type 2 diabetes. Applied Physiology, Nutrition and Metabolism, 45(3), 283–293. 10.1139/apnm-2019-0208 PMC12548805 31356754

[phy214681-bib-0016] Gunnarsson, T. P. , & Bangsbo, J. (2012). The 10–20‐30 training concept improves performance and health profile in moderately trained runners. Journal of Applied Physiology, 113(1), 16–24. 22556401 10.1152/japplphysiol.00334.2012

[phy214681-bib-0017] Hatou, S. , Yamada, M. , Akune, Y. , Mochizuki, H. , Shiraishi, A. , Joko, T. , Nishida, T. , & Tsubota, K. (2010). Role of insulin in regulation of Na+‐/K+‐dependent ATPase activity and pump function in corneal endothelial cells. Investigative Ophthalmology & Visual Science, 51(8), 3935–3942. 20335606 10.1167/iovs.09-4027

[phy214681-bib-0018] He, J. , Watkins, S. , & Kelley, D. E. (2001). Skeletal muscle lipid content and oxidative enzyme activity in relation to muscle fiber type in type 2 diabetes and obesity. Diabetes, 50(4), 817–823. 11289047 10.2337/diabetes.50.4.817

[phy214681-bib-0019] Henriksen, E. J. , Diamond‐Stanic, M. K. , & Marchionne, E. M. (2011). Oxidative stress and the etiology of insulin resistance and type 2 diabetes. Free Radical Biology and Medicine, 51(5), 993–999. 21163347 10.1016/j.freeradbiomed.2010.12.005 PMC3071882

[phy214681-bib-0020] Hernandez‐Alvarez, M. I. , Thabit, H. , Burns, N. , Shah, S. , Brema, I. , Hatunic, M. , Finucane, F. , Liesa, M. , Chiellini, C. , Naon, D. , Zorzano, A. , & Nolan, J. J. (2010). Subjects with early‐onset type 2 diabetes show defective activation of the skeletal muscle PGC‐1{alpha}/Mitofusin‐2 regulatory pathway in response to physical activity. Diabetes Care, 33(3), 645–651. 20032281 10.2337/dc09-1305 PMC2827524

[phy214681-bib-0021] Hostrup, M. , & Bangsbo, J. (2017). Limitations in intense exercise performance of athletes – Effect of speed endurance training on ion handling and fatigue development. Journal of Physiology, 595(9), 2897–2913. 10.1113/JP273218 PMC5407979 27673449

[phy214681-bib-0022] Iaia, F. M. , Thomassen, M. , Kolding, H. , Gunnarsson, T. , Wendell, J. , Rostgaard, T. , Nordsborg, N. , Krustrup, P. , Nybo, L. , Hellsten, Y. , & Bangsbo, J. (2008). Reduced volume but increased training intensity elevates muscle Na+‐K+ pump alpha1‐subunit and NHE1 expression as well as short‐term work capacity in humans. American Journal of Physiology: Regulatory, Integrative and Comparative Physiology, 294(3), R966–R974. 10.1152/ajpregu.00666.2007 18094063

[phy214681-bib-0023] Jheng, H. F. , Tsai, P. J. , Guo, S. M. , Kuo, L. H. , Chang, C. S. , Su, I. J. , Chang, C.‐R. , & Tsai, Y.‐S. (2012). Mitochondrial fission contributes to mitochondrial dysfunction and insulin resistance in skeletal muscle. Molecular and Cellular Biology, 32(2), 309–319. 22083962 10.1128/MCB.05603-11 PMC3255771

[phy214681-bib-0024] Juel, C. , Holten, M. K. , & Dela, F. (2004). Effects of strength training on muscle lactate release and MCT1 and MCT4 content in healthy and type 2 diabetic humans. Journal of Physiology, 556(Pt 1), 297–304. 10.1113/jphysiol.2003.058222 PMC1664883 14724187

[phy214681-bib-0025] Kjeldsen, K. , Braendgaard, H. , Sidenius, P. , Larsen, J. S. , & Norgaard, A. (1987). Diabetes decreases Na+‐K+ pump concentration in skeletal muscles, heart ventricular muscle, and peripheral nerves of rat. Diabetes, 36(7), 842–848. 3034710 10.2337/diab.36.7.842

[phy214681-bib-0026] Larsen, S. , Nielsen, J. , Hansen, C. N. , Nielsen, L. B. , Wibrand, F. , Stride, N. , Schroder, H. D. , Boushel, R. , Helge, J. W. , Dela, F. , & Hey‐Mogensen, M. (2012). Biomarkers of mitochondrial content in skeletal muscle of healthy young human subjects. Journal of Physiology, 590(14), 3349–3360. 10.1113/jphysiol.2012.230185 PMC3459047 22586215

[phy214681-bib-0027] Li, R. , & Shen, Y. (2013). An old method facing a new challenge: re‐visiting housekeeping proteins as internal reference control for neuroscience research. Life Sciences, 92(13), 747–751. 23454168 10.1016/j.lfs.2013.02.014 PMC3614345

[phy214681-bib-0028] Little, J. P. , Gillen, J. B. , Percival, M. E. , Safdar, A. , Tarnopolsky, M. A. , Punthakee, Z. , Jung, M. E. , & Gibala, M. J. (2011). Low‐volume high‐intensity interval training reduces hyperglycemia and increases muscle mitochondrial capacity in patients with type 2 diabetes. Journal of Applied Physiology, 111(6), 1554–1560. 21868679 10.1152/japplphysiol.00921.2011

[phy214681-bib-0029] Lowell, B. B. , & Shulman, G. I. (2005). Mitochondrial dysfunction and type 2 diabetes. Science, 307(5708), 384–387. 15662004 10.1126/science.1104343

[phy214681-bib-0030] Lowry, O. H. , & Passonneau, J. V. (1972). A flexible system of enzymatic analysis (Vol. xii, 291 p.). Academic Press.

[phy214681-bib-0031] Murphy, R. M. , & Lamb, G. D. (2013). Important considerations for protein analyses using antibody based techniques: down‐sizing Western blotting up‐sizes outcomes. Journal of Physiology, 591(23), 5823–5831. 10.1113/jphysiol.2013.263251 PMC3872754 24127618

[phy214681-bib-0032] Nishida, K. , Ohara, T. , Johnson, J. , Wallner, J. S. , Wilk, J. , Sherman, N. , Kawakami, K. , Sussman, K. E. , & Draznin, B. (1992). Na+/K(+)‐ATPase activity and its alpha II subunit gene expression in rat skeletal muscle: influence of diabetes, fasting, and refeeding. Metabolism, 41(1), 56–63. 1311403 10.1016/0026-0495(92)90191-c

[phy214681-bib-0033] Odegaard, A. O. , Jacobs, D. R. Jr , Sanchez, O. A. , Goff, Jr D. C. , Reiner, A. P. , & Gross, M. D. (2016). Oxidative stress, inflammation, endothelial dysfunction and incidence of type 2 diabetes. Cardiovascular Diabetology, 15, 51. 27013319 10.1186/s12933-016-0369-6 PMC4806507

[phy214681-bib-0034] Pan, H. Z. , Zhang, L. , Guo, M. Y. , Sui, H. , Li, H. , Wu, W. H. , Nai‐qiang, Q. U. , Liang, M.‐H. , & Chang, D. (2010). The oxidative stress status in diabetes mellitus and diabetic nephropathy. Acta Diabetologica, 47(Suppl. 1), 71–76. 19475334 10.1007/s00592-009-0128-1

[phy214681-bib-0035] Perry, C. G. , Lally, J. , Holloway, G. P. , Heigenhauser, G. J. , Bonen, A. , & Spriet, L. L. (2010). Repeated transient mRNA bursts precede increases in transcriptional and mitochondrial proteins during training in human skeletal muscle. Journal of Physiology, 588(Pt 23), 4795–4810. 10.1113/jphysiol.2010.199448 PMC3010147 20921196

[phy214681-bib-0036] Persson, M. , Steinz, M. M. , Westerblad, H. , Lanner, J. T. , & Rassier, D. E. (2019). Force generated by myosin cross‐bridges is reduced in myofibrils exposed to ROS/RNS. American Journal of Physiology. Cell Physiology. 10.1152/ajpcell.00272.2019 31553646

[phy214681-bib-0037] Phielix, E. , Meex, R. , Moonen‐Kornips, E. , Hesselink, M. K. , & Schrauwen, P. (2010). Exercise training increases mitochondrial content and ex vivo mitochondrial function similarly in patients with type 2 diabetes and in control individuals. Diabetologia, 53(8), 1714–1721. 20422397 10.1007/s00125-010-1764-2 PMC2892060

[phy214681-bib-0038] Ramakrishna, V. , & Jailkhani, R. (2008). Oxidative stress in non‐insulin‐dependent diabetes mellitus (NIDDM) patients. Acta Diabetologica, 45(1), 41–46. 17924055 10.1007/s00592-007-0018-3

[phy214681-bib-0039] Regensteiner, J. G. , Bauer, T. A. , Reusch, J. E. , Brandenburg, S. L. , Sippel, J. M. , Vogelsong, A. M. , Smith, S. , Wolfel, E. E. , Eckel, R. , & Hiatt, W. R. (1998). Abnormal oxygen uptake kinetic responses in women with type II diabetes mellitus. Journal of Applied Physiology, 85(1), 310–317. 9655791 10.1152/jappl.1998.85.1.310

[phy214681-bib-0040] Reusch, J. E. B. , Bridenstine, M. , & Regensteiner, J. G. (2013). Type 2 diabetes mellitus and exercise impairment. Reviews in Endocrine and Metabolic Disorders, 14(1), 77–86. 23299658 10.1007/s11154-012-9234-4 PMC3593997

[phy214681-bib-0041] Rognmo, O. , Moholdt, T. , Bakken, H. , Hole, T. , Molstad, P. , Myhr, N. E. , Grimsmo, J. , & Wisløff, U. (2012). Cardiovascular risk of high‐ versus moderate‐intensity aerobic exercise in coronary heart disease patients. Circulation, 126(12), 1436–1440. 22879367 10.1161/CIRCULATIONAHA.112.123117

[phy214681-bib-0042] Rovira‐Llopis, S. , Banuls, C. , Diaz‐Morales, N. , Hernandez‐Mijares, A. , Rocha, M. , & Victor, V. M. (2017). Mitochondrial dynamics in type 2 diabetes: Pathophysiological implications. Redox Biology, 11, 637–645. 28131082 10.1016/j.redox.2017.01.013 PMC5284490

[phy214681-bib-0043] Sarigianni, M. , Tsapas, A. , Mikhailidis, D. P. , Kaloyianni, M. , Koliakos, G. , & Paletas, K. (2010). Involvement of signaling molecules on na/h exchanger‐1 activity in human monocytes. Open Cardiovascular Medicine Journal, 4, 181–188. 10.2174/1874192401004010181 PMC3002055 21160910

[phy214681-bib-0044] Schmidt, T. A. , Hasselbalch, S. , Farrell, P. A. , Vestergaard, H. , & Kjeldsen, K. (1994). Human and rodent muscle Na(+)‐K(+)‐ATPase in diabetes related to insulin, starvation, and training. Journal of Applied Physiology, 76(5), 2140–2146. 8063678 10.1152/jappl.1994.76.5.2140

[phy214681-bib-0045] Sweeney, G. , & Klip, A. (1998). Regulation of the Na+/K+‐ATPase by insulin: Why and how? Molecular and Cellular Biochemistry, 182(1–2), 121–133. 9609121

[phy214681-bib-0046] Szendroedi, J. , Phielix, E. , & Roden, M. (2011). The role of mitochondria in insulin resistance and type 2 diabetes mellitus. Nature Reviews Endocrinology, 8(2), 92–103. 10.1038/nrendo.2011.138 21912398

[phy214681-bib-0047] Thomassen, M. , Hostrup, M. , Murphy, R. M. , Cromer, B. A. , Skovgaard, C. , Gunnarsson, T. P. , Christensen, P. M. , & Bangsbo, J. (2018). Abundance of ClC‐1 chloride channel in human skeletal muscle: Fiber type specific differences and effect of training. Journal of Applied Physiology, 125(2), 470–478. 29722626 10.1152/japplphysiol.01042.2017

[phy214681-bib-0048] Timmons, J. A. , Atherton, P. J. , Larsson, O. , Sood, S. , Blokhin, I. O. , Brogan, R. J. , Volmar, C.‐H. , Josse, A. R. , Slentz, C. , Wahlestedt, C. , Phillips, S. M. , Phillips, B. E. , Gallagher, I. J. , & Kraus, W. E. (2018). A coding and non‐coding transcriptomic perspective on the genomics of human metabolic disease. Nucleic Acids Research, 46(15), 7772–7792. 29986096 10.1093/nar/gky570 PMC6125682

[phy214681-bib-0049] Tjonna, A. E. , Lee, S. J. , Rognmo, O. , Stolen, T. O. , Bye, A. , Haram, P. M. , Loennechen, J. P. , Al‐Share, Q. Y. , Skogvoll, E. , Slørdahl, S. A. , Kemi, O. J. , Najjar, S. M. , & Wisløff, U. (2008). Aerobic interval training versus continuous moderate exercise as a treatment for the metabolic syndrome: A pilot study. Circulation, 118(4), 346–354. 18606913 10.1161/CIRCULATIONAHA.108.772822 PMC2777731

[phy214681-bib-0050] Wilson, M. C. , Jackson, V. N. , Heddle, C. , Price, N. T. , Pilegaard, H. , Juel, C. , Bonen, A. , Montgomery, I. , Hutter, O. F. , & Halestrap, A. P. (1998). Lactic acid efflux from white skeletal muscle is catalyzed by the monocarboxylate transporter isoform MCT3. Journal of Biological Chemistry, 273(26), 15920–15926. 10.1074/jbc.273.26.15920 9632638

[phy214681-bib-0051] Wohaieb, S. A. , & Godin, D. V. (1987). Alterations in free radical tissue‐defense mechanisms in streptozocin‐induced diabetes in rat. Effects of Insulin Treatment. Diabetes, 36(9), 1014–1018. 3301471 10.2337/diab.36.9.1014

